# Aggregation and Sedimentation of *Thalassiosira weissflogii* (diatom) in a Warmer and More Acidified Future Ocean

**DOI:** 10.1371/journal.pone.0112379

**Published:** 2014-11-06

**Authors:** Shalin Seebah, Caitlin Fairfield, Matthias S. Ullrich, Uta Passow

**Affiliations:** 1 Molecular Life Science Research Center, Jacobs University Bremen, Bremen, Germany; 2 Marine Science Institute, University of California Santa Barbara, Santa Barbara, California, United States of America; Scottish Association for Marine Science, United Kingdom

## Abstract

Increasing Transparent Exopolymer Particle (TEP) formation during diatom blooms as a result of elevated temperature and *p*CO_2_ have been suggested to result in enhanced aggregation and carbon flux, therewith potentially increasing the sequestration of carbon by the ocean. We present experimental results on TEP and aggregate formation by *Thalassiosira weissflogii* (diatom) in the presence or absence of bacteria under two temperature and three *p*CO_2_ scenarios. During the aggregation phase of the experiment TEP formation was elevated at the higher temperature (20°C vs. 15°C), as predicted. However, in contrast to expectations based on the established relationship between TEP and aggregation, aggregation rates and sinking velocity of aggregates were depressed in warmer treatments, especially under ocean acidification conditions. If our experimental findings can be extrapolated to natural conditions, they would imply a reduction in carbon flux and potentially reduced carbon sequestration after diatom blooms in the future ocean.

## Introduction

Globally, gravitational sinking of marine snow (>0.5 mm) contributes significantly to the biological carbon pump, leading to carbon sequestration into the deep ocean. Large sedimentation events are frequently associated with diatom blooms, because most bloom- forming diatoms form marine snow-sized aggregates. Coagulation of diatoms is impacted by diatom species [1], bacteria species and activities [2]–[4], and extrapolymeric substances (EPS) [5], [6], especially transparent exopolymer particles (TEP) [7]–[9] and may be described using aggregation theory [10]–[12].

Atmospheric *p*CO_2_ values are expected to rise to a global average of 750 ppm (IPCC scenario IS92a, IPCC 2007) and perhaps even beyond 1000 ppm by the end of this century [13]. Increasing atmospheric CO_2_ concentrations do not solely result in higher sea-surface temperatures due to intensified radiative forcing, but also lead to ocean acidification [14]. The term ocean acidification describes the increase in dissolved inorganic carbon (DIC) and the concomitant decrease in pH in surface waters [15]. Changing oceanic conditions due to globally rising temperatures and ocean acidification may influence the functioning of the biological pump and its specific responses to these changes are currently under intense investigation [16].

The potential increase in TEP concentration as a result of elevated temperature and *p*CO_2_ has been suggested to result in enhanced aggregation and flux [17], [18], although this finding has also been challenged [19]. Reduced production of coccoliths under ocean acidification conditions is thought to reduce sedimentation of carbon due to a reduction in ballasting [20]–[24]. Coagulation of organic matter with lithogenic minerals has not been found to be impacted by ocean acidification [25].

Here, the combined effects of changed carbonate chemistry and temperature on the coagulation of axenic and xenic cultures of the diatom *Thalassiosira weissflogii* were investigated. Xenic cultures contained the marine gammaproteobacterium *Marinobacter adhaerens* HP15, which has been shown to promote TEP production and induce aggregation of the diatom *Thalassiosira weissflogii* [2]. Aggregation, TEP concentration and sinking velocities of aggregates were monitored during the four-day aggregation experiments.

## Materials and Methods

### Experimental design and set up

The combined impact of temperature, ocean acidification and bacterial activity on the formation of TEP and aggregates by the diatom *Thalassiosira weissflogii* was tested in a full factorial design. The established bilateral model system between the diatom *T. weissflogii* and the marine bacterium *M. adhaerens* HP15 [26], [27] was used to investigate the bacterial contribution to TEP formation and aggregation under the different environmental conditions. Three different carbonate chemistry regimes were selected to reflect: (i) the present-day conditions, with the partial pressure of CO_2_ (*p*CO_2_) ranging between 300–350 µatm (termed Ambient) and (ii) two future ocean scenarios with *p*CO_2_ ranging from 750–850 µatm (designated Future 1) and 1000–1250 µatm (referred to as Future 2). For each carbonate chemistry regime, two temperatures were chosen, 15°C and 20°C (Table 1).

**Table 1:** Design of multifactorial experiment with 12 treatments testing aggregation of the diatom *T. weissflogii* in the presence or absence of bacteria at two temperatures and three *p*CO_2_ scenarios.

Treat. #	Temp. °C	*p*CO_2_	Bact.
1	15	Am	Ax
2	15	Am	HP
3	15	F1	Ax
4	15	F1	HP
5	15	F2	Ax
6	15	F2	HP
7	20	Am	Ax
8	20	Am	HP
9	20	F1	Ax
10	20	F1	HP
11	20	F2	Ax
12	20	F2	HP

Xenic treatments contained the bacterium *M. adhaerens* HP15. Each treatment was prepared in triplicate; one replicate was harvested initially (t = 0) and two after 96 hr. incubation on roller tables in the dark. See text for specifics on *p*CO_2_ treatments. Ax  =  axenic, HP  =  *M. adhaerens* HP15 added, Am  =  Ambient, F1  =  Future 1, F2  =  Future 2.

An axenic diatom culture of *Thalassiosira weissflogii* (CCMP 1336) and the bacterium *M. adhaerens* HP15 were used for experiments. *M. adhaerens* HP15 was isolated from marine particles collected from the surface waters of the German Bight (Grossart et al. 2004). *M. adhaerens* HP15 attaches preferentially to *T.weissflogii* cells and impacts TEP production and aggregation [2].

Due to logistical reasons, the experiment was run in two sections. First the six treatments at 15°C, and a few days later the six treatments at 20°C were incubated in duplicate rolling tanks and in darkness at three rotations per minute (rpm). Solid body rotation was established in roller tanks (1.15-L Plexiglas cylinders with a diameter of 14 cm and a depth of 7.47 cm) within 2–3 hours, which assured that aggregates remained suspended, never contacting container walls [28]. Incubation in roller tanks in the dark mimicked sinking of aggregates through the water column to depth. The experiment was terminated after 96 hrs. Prior to the experiment, the diatom and bacterial cultures were separately acclimatized to the respective temperature and carbonate chemistry regimes for more than 8 generations to avoid a stress reaction to changed environmental conditions.

During the acclimatization phase diatoms were grown at 50 µE s^−1^ for a 12-hr light period, in a semi-continuous batch approach, to ensure continuous exponential growth and restrict changes in the carbonate system. Diatom numbers and total alkalinity (TA), pH and DIC were monitored daily during this phase and cultures diluted (factor 2–6) before a cell concentration of 60, 000 cells mL^−1^ was reached or the pH changed by more than 0.25 units. *M. adhaerens* HP15 was acclimatized to the respective temperature and *p*CO_2_ conditions overnight in sterile culture flasks with aeration of approximately 250 rpm.

After the acclimatization phase, triplicate roller tanks were filled bubble-free under sterile conditions with diatom cells at a final concentration of 3×10^3^ cells mL^−1^ and bacterial cells at a final concentration of 3×10^5^ cells mL^−1^. Diatom blooms in coastal and upwelling areas regularly reach cell concentrations of 10^4^ cells mL^−1^, when small diatoms dominate and may reach 10^5^ cells mL^−1^, for example in the upwelling area of the Benguela current or off the California coast [29], [30]. The chosen diatom concentration is thus still ecologically relevant while providing enough cells to allow rapid aggregation and provide enough aggregates for the required measurements. Prior to inoculation with the diatom culture, the bacterial cells were washed twice in sterile seawater to minimize carry-over of nutrients or bacterial growth-derived matter into the ASW media. One replicate roller tank per treatment was sacrificed at the beginning and two replicates per treatment at the end of the experiment. TEP concentration, and aggregate size, number, and sinking velocity, as well as the carbonate system parameters (TA, pH and DIC) were analyzed at both time points. Values are given as averages ± standard deviation of the duplicate tanks, with standard deviations calculated using error propagation, where appropriate.

Aggregates were defined as particles ≥0.5 mm. During sampling, aggregates, when present, were first removed using a cut-off pipette [31], their sinking velocities determined and all aggregates of one tank combined in a known volume of artificial seawater, creating aggregate slurry. The surrounding seawater (SSW), which remained in the tank after the manual removal of the aggregates, was sampled thereafter. TEP was measured in aggregate slurries and in the SSW; the carbonate system parameters were determined in the SSW.

### Cultures and media

Autoclaving of natural seawater (collected off Santa Barbara 34° 23′ N 119° 50′ W) for media preparation was not an option since the carbonate chemistry of seawater is severely impacted by de-gassing. The pH of freshly collected natural seawater from the Santa Barbara Channel increased from 7.58 to 8.67 during autoclaving. Stirring the autoclaved seawater while leaving the beaker open to the atmosphere only reduced the pH to 7.89 (Table 2). The repeated filtration of natural seawater through 0.2 µm pore-sized filters (Millipore, MA, USA) did not satisfactorily remove all bacterial contaminants. We therefore opted for the use of artificial seawater (ASW) [32] for media preparation. Using ASW imparts the added benefit of easily and precisely manipulating DIC concentrations. ASW was prepared with a DIC concentration of 2,050 µmol kg^−1^ for ambient treatments and supplemented with vitamins and trace metal solutions as in F/2 medium [33]. Macronutrients were added to a final concentration of 59 µM nitrate, 3.6 µM phosphate and 53.5 µM silicic acid to create ASW-media. The carbonate chemistry of future treatments was adjusted as described below.

**Table 2:** Effect of autoclaving on the carbonate chemistry of seawater.

Sample	pH (total)
Fresh seawater before autoclaving	7.58
After autoclaving (with or without nutrients)	8.66±0.01
after stirring for 24 hrs.	8.39
after stirring for 72 hrs.	7.94
after stirring for 96 hrs.	7.90
after stirring for 120 hrs.	7.89

Diatom cells were counted in a Sedgwick-Rafter Cell S50 (SPI Supplies, West Chester, PA, USA) using an inverted Axiovert 200 microscope (Zeiss, Jena, Germany). The axenicity of the diatom culture was checked by epifluorescence microscopy [34] after staining with the dye 4′, 6-diamidino-2-phenylindol (DAPI) [35].

### Carbonate chemistry perturbations and analysis

Since TEP production has been shown to be impacted by bubbling [36]–[38], the carbonate system was chemically perturbed as described in Passow [25], [39]. Briefly, to mimic future ocean conditions, appropriate amounts of 0.1 M HCl (mL kg^−1^), 0.1 M NaHCO_3_ (mL kg^−1^) and 0.001 M Na_2_CO_3_ (mL kg^−1^) were added to change DIC and pH while keeping TA constant. Measurements of pH and TA confirmed that our perturbations changed the system as expected and reflected those anticipated in a future ocean.

The carbonate system was monitored by measuring pH, TA and DIC. Samples for pH were collected bubble-free in 20-mL scintillation vials and the pH (total scale, pH_T_) was measured with a spectrophotometer using the indicator dye m-cresol purple (Sigma-Aldrich) within 2 hours of sampling. The measurement temperature was held at 25°C and the absorbance measured at 730 nm, 578 nm and 434 nm before and after dye addition [40], [41]. The pH was calculated following the standard operating procedure (SOP 7) [42]. Samples for TA and DIC measurements were collected following SOP 1 [42]. TA and DIC samples were poisoned with 0.02% saturated HgCl_2_ by volume and sent for analysis to the Dickson Laboratory at the Scripps Institution of Oceanography, UCSD.

The program CO_2_ Sys [43] was used to calculate the carbonate system. The dissociation constants K1 and K2 from Roy [44] were used since these have been described as the most appropriate for ASW [15]. Any two of the main carbonate parameters (pH, TA, DIC, *p*CO_2_) describe the carbonate system sufficiently and the other parameters can be calculated from the measured ones. In 50 different samples, we measured three carbonate parameters (pH, TA and DIC) to over-determine the carbonate system.

### Quantification of TEP

TEP concentrations were measured colorimetrically by filtration of samples onto 0.4-µm pore size polycarbonate filters (Millipore, MA, USA) and subsequent staining with Alcian blue [45]. TEP concentration was determined in quadruplicate and expressed as Gum Xanthan equivalents per liter (GXeq L^−1^).

### Aggregate number, size and sinking velocity

The number of aggregates >0.5 mm was counted and the sinking velocity of 10 to 20 aggregates per tank per treatment measured by gently transferring individual aggregates from the roller tanks to a tall 1 liter cylinder containing sterile ASW media [28]. Prior to measuring sinking velocity of aggregates, the ASW medium was incubated overnight at either 15°C or 20°C to ensure that aggregates experienced no change in environmental conditions during the sinking velocity determinations. The time taken for each aggregate to sink 25 cm was recorded. The dimensions of the aggregate axes (x, y, and z direction) were measured under a dissecting microscope, using grid paper and the aggregated volume was calculated by assuming an ellipsoid shape. The equivalent spherical diameter (ESD) was calculated. Sinking velocity could only be measured on 5–8 aggregates for some treatments, because of the lack of aggregates. The slopes of the sinking velocity vs. aggregate size relationships at 15°C and 20°C were compared by calculating both pooled and unpooled error variance and the appropriate t and p values.

No specific permissions were required to collect seawater samples off Santa Barbara and no endangered or protected species were used for our experiments. The data of this study is deposited at the Oceanographic Data Repository BCO-DMO (Biological and Chemical Oceanography Data Management Office; http://www.bco-dmo.org/) in accordance with NSF guidelines. Doi: 10.1575/1912/6845; http://hdl.handle.net/1912/6845.

## Results

### Over-determination of the carbonate chemistry

**Figure 1:**
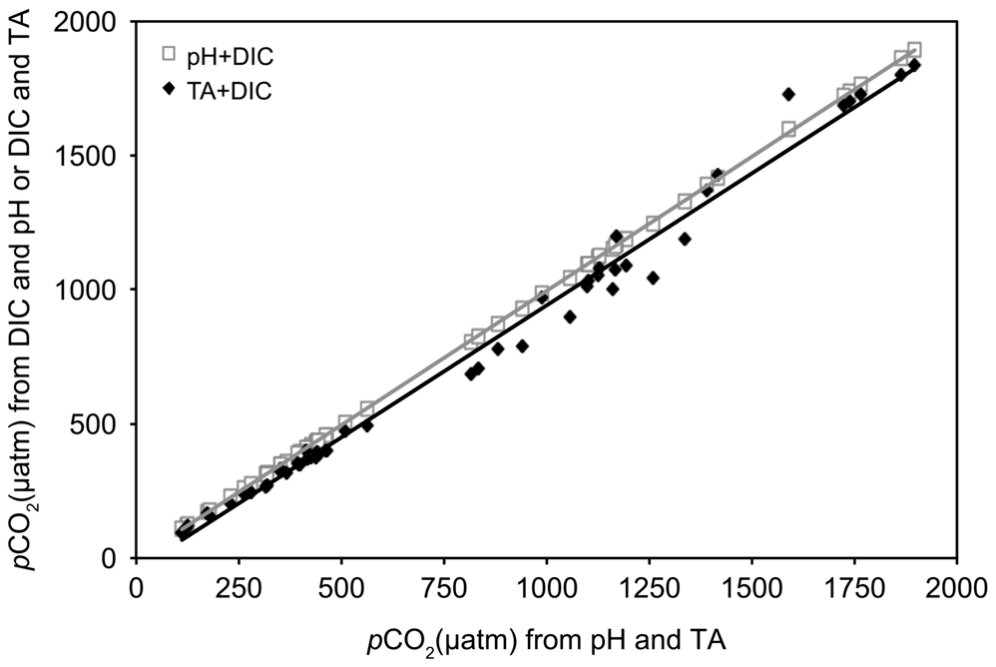
Relationship between *p*CO_2_ determined from DIC and pH or TA vs. *p*CO_2_ determined from pH and TA. Data stems from 50 random samples from the experiment, where the carbonate chemistry was over-determined.

The *p*CO_2_ concentrations in samples where the carbonate system was over-determined were calculated using all three possible carbonate parameter combinations. The slight but consistent discrepancy in the *p*CO_2_ concentrations depending on the parameter combination used for calculation (Fig. 1) is well known and has been described [39], [46]. Results from over-determined carbonate system parameters confirm that our measurements were consistent and all treatments exposed to targeted conditions.

### Acclimatization phase

By frequent dilutions of the diatom cultures with media adjusted to the appropriate pH and TA, it was possible to maintain the carbonate system of the cultures within a narrow range during the acclimatization phase. The average TA was 2332±32 µmol kg^−1^ and did not significantly vary statistically between any of the treatments (p>0.05). The maximal temporal shift in pH experienced in each *p*CO_2_ treatment due to growth of phytoplankton was kept to <0.3 pH units, usually <0.2 units (Table 3). In coastal upwelling systems or during phytoplankton blooms, *in situ* variations in pH may easily be that large. For example, off California daily ranges of pH are frequently 0.2 to 0.3 units [47], [48]. *T. weissflogii* were acclimatized 8 and 11 days, depending on growth rate. Exponential growth rates were a significant function of temperature, but not *p*CO_2_, although growth rates were slightly decreased under Future 2 conditions at both temperatures (Table 3).

**Table 3:** Exponential growth of *T. weissflogii* and pH range during the acclimatization phase.

	Treatment	µ (d^−1^)	pH_T_	No. of days acclimatized
15°C	Ambient	0.51	7.93–8.21	11
	Future 1	0.52	7.57–7.76	11
	Future 2	0.49	7.45–7.66	11
20°C	Ambient	0.86	8.04–8.23	8
	Future 1	0.86	7.61–7.84	8
	Future 2	0.82	7.46–7.67	8

### Aggregation experiment

#### Carbonate system

**Figure 2:**
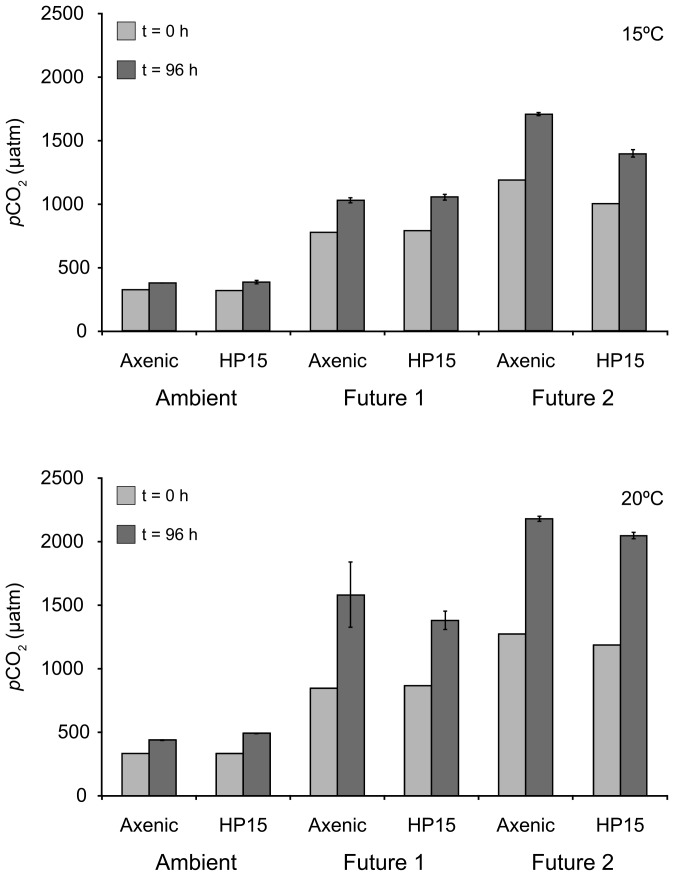
Initial and final *p*CO_2_ during the incubations at 15°C and 20°C.

The average TA was 2351±7 µmol kg^−1^ in all treatments. Initial Ambient pH_T_ at *in vitro* temperature was 8.15±0.00 and 8.14±0.00 in 15°C and 20°C treatments, respectively. The initial pH_T_ in Future 1 treatments were 0.34±0.01 (15°C treatments) and 0.35±0.01 (20°C treatments) units lower than the respective Ambient treatments, while those of Future 2 were 0.47±0.05 (15°C treatments) and 0.50±0.02 (20°C treatments) units lower. The associated initial *p*CO_2_ values for Ambient, Future 1 and Future 2 were 294±1 µatm; 722±7 µatm and 1021±123 µatm in 15°C treatments and 304±2 µatm, 782±14 µatm and 1139±55 µatm in 20°C treatments (Fig. 2).

During the 96 hr. incubation in rolling tanks, the pH_T_ dropped between 0.06 and 0.22 units, with the largest temporal change in the two Future treatments at 20°C, reflecting the combination of higher respiration rates at higher temperatures and a weaker buffering system under future carbonate chemistry conditions. The simultaneous temporal change in *p*CO_2_ ranged from 54 to 840 µatm, with the final *p*CO_2_ in the 20°C Future 2 treatment reaching almost 2000 µatm (Fig. 2).

#### TEP formation

TEP were inadvertently added to each treatment with the diatom inoculum, and differences in initial concentrations reflect differences in TEP concentrations after the acclimatization phase. Initial TEP concentrations in the 12 treatments ranged between 356 µg GXeq. L^−1^ and 1148 µg GXeq. L^−1^, with significantly higher initial TEP concentrations in the six treatments incubated at 15°C compared to those at 20°C treatments (Mann-Whitney U-test, p<0.05).

**Figure 3:**
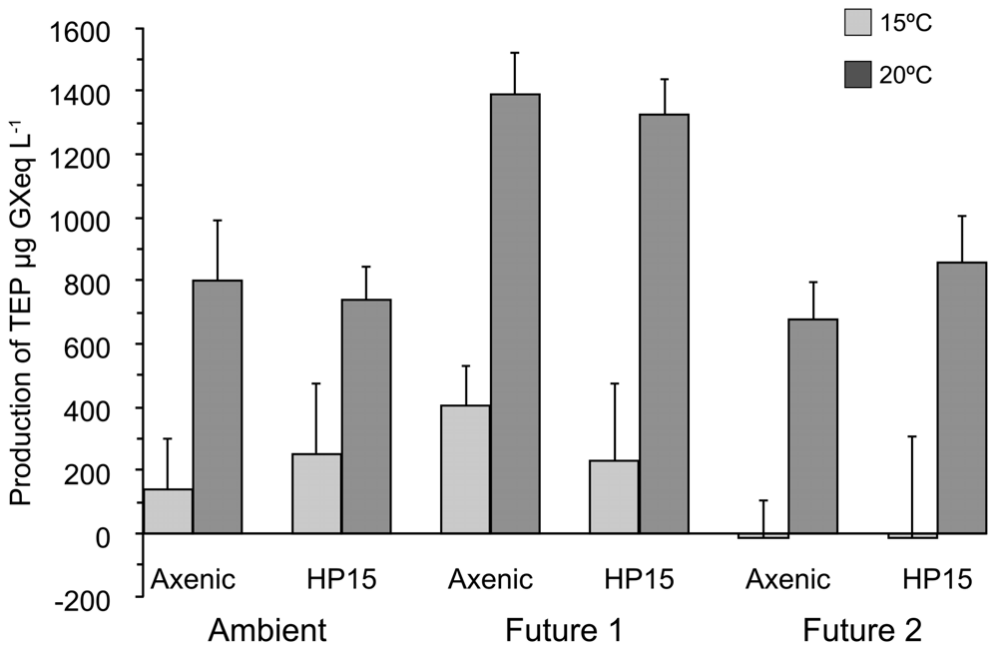
Production of Transparent Exopolymer Particles (TEP) during the incubations in all treatments, calculated as net change during the 96 hrs. experiment, and errors calculated using error propagation.

During the 96 hr. incubation TEP remained about constant or increased moderately in treatments at 15°C, whereas the increase was appreciably higher in all treatments that were incubated at 20°C (Fig. 3). As a result, average TEP production of all 20°C treatments was significantly higher than that in 15°C treatments (Table 4), irrespective of *p*CO_2_ conditions or the presence of bacteria (Mann-Whitney U-test, p<0.05). The presence of *M. adhaerens* HP15 had no effect on the amount of TEP generated during the experiment; TEP production averaged 565 µg GXeq L^−1^ with or without bacteria (Table 4). The *p*CO_2_ conditions also did not influence TEP production significantly (Table 4; (Mann-Whitney U-test, p>0.05), but variability was high: Within each temperature the highest production of TEP was observed under Future 1 conditions, suggesting the possibility of some, albeit non-linear and complex impact of *p*CO_2_, on TEP production. However, this could not be resolved with our experimental set-up.

**Table 4:** Comparison of average TEP production (µg GXeq. L^−1^) and aggregation, as measured by total aggregate volume (Agg. Vol.), combining treatments with the same temperature, carbonate conditions, or state of axenicity, respectively.

Treatment	TEP production µg GXeq. L^−1^	Total Agg. Vol. cm3	n
15°C	166±164*	2.04±0.60*	6
20°C	965±311*	0.82±0.73*	6
Am	483±335	2.01±0.74	4
F1	837±605	1.21±1.15	4
F2	376±458	1.07±0.66	4
Ax	565±508	1.56±0.95	6
HP	565±498	1.30±0.92	6

N =  number of treatments, each in duplicate.

*: averages significantly (p<0.05) different from each other, paired t-test.

Between 23 and 50% of all TEP was incorporated in aggregates (Table 5). Although the fraction of TEP in aggregates was always higher in the 20°C treatments compared to the 15°C treatments (34±9% vs. 26±3%), this trend was too small compared to the high variability to be statistically significant. The fraction of TEP enclosed in aggregates was largest under Ambient conditions (36±10%) and smallest under Future 2 conditions (25±3%). The partitioning of TEP between the aggregated and un-aggregated phase was independent of TEP concentration and the presence of bacteria.

**Table 5:** TEP in Aggregates, absolute amount and fraction.

Treatment	15°C		20°C	
	GXeq. L^−1^	%	GXeq. L^−1^	%
Am HP	410	31	432	36
Am Ax	280	26	659	50
F1 HP	319	26	471	28
F1 Ax	299	24	641	35
F2 HP	260	23	349	24
F2 Ax	260	25	381	29

### Aggregation

**Figure 4:**
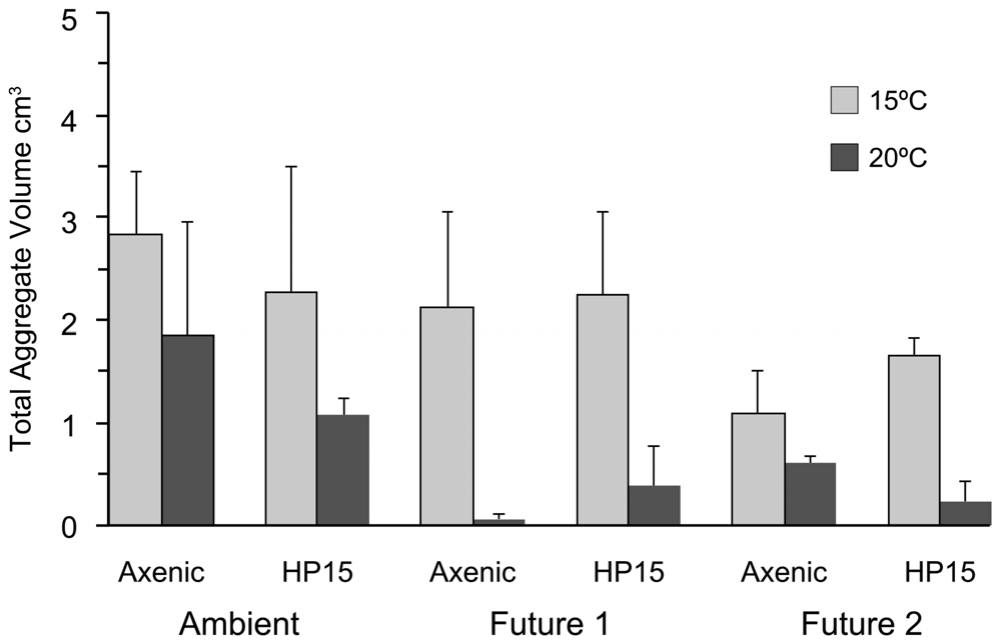
Total aggregate volume after the incubations in all treatments; error bars represent the range of replicates.

Between 2 and 36 aggregates formed in the tanks, with significantly fewer aggregates in 20°C treatments compared to the 15°C treatments (Mann-Whitney U-test, p<0.05). Total aggregate volume, which is a better indicator of aggregate formation because it combines size and abundance of aggregates, was also significantly smaller in 20°C treatments compared to 15°C treatments. Total aggregate volume was high (>1 cm^3^) in all treatments at 15°C, as well as in both ambient treatments at 20°C (Fig. 4). In contrast, total aggregate volume was small (<0.7 cm^3^) in all four future treatments at 20°C, independent of the presence or absence of bacteria. Total aggregate volume was not a significant function of total TEP concentration. Not only was the relationship not significant, but higher TEP concentration tended to result in a smaller total aggregate volume.

### Sinking velocity

**Figure 5:**
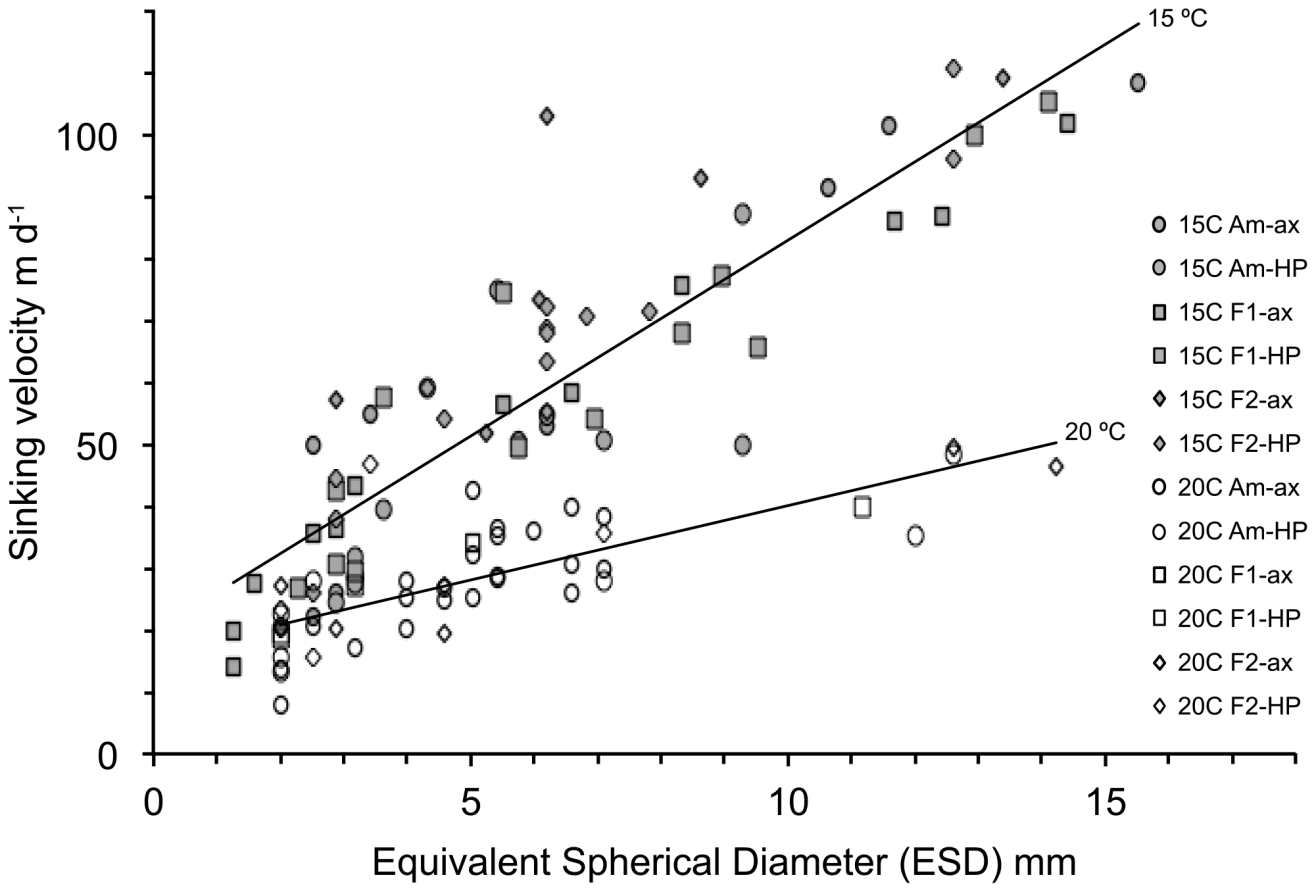
Sinking velocity vs. size (equivalent spherical diameter) of aggregates >0.5 mm that formed in the different treatments. Lines represent the regression of aggregates incubated at 15°C and 20°C, respectively.

Measured sinking velocities of aggregates >0.5 mm ranged between 8 and 110 m d^−1^. Sinking velocity increased with the size of aggregates (equivalent spherical diameter, ESD), but was independent of *p*CO_2_ or bacterial presence (Fig. 5). The slope of the velocity-size relationship depended on treatment (Table 6) and the slope of the sinking velocity vs. size relationship was significantly smaller (p<0.001, df = 111, slope-t-test) for the 20°C treatments (*y* = 2.4(±0.3)*x*+16.1(±1.8), df = 44, r^2^ = 0.59) compared to 15°C treatments (*y* = 6.3(±0.4)*x*+19.7(±2.8), df = 67, r^2^ = 0.80) (Fig. 5). This resulted in relatively low sinking velocities, especially of larger aggregates, in 20°C treatments: For example, an aggregate with an ESD of 7 mm sank with 60 m d^−1^ in 15°C treatments and with 30 m d^−1^ in 20°C treatments.

**Table 6:** Slopes of sinking velocity vs. equivalent spherical diameter (ESD) regressions in each treatment (Amb  =  Ambient, F1and F2  =  Future 1 and Future 2, Ax  =  axenic, HP  =  *M. adhaerens* HP15).

Treatment	slope	n	r^2^
15 Amb-Ax	6.5	13	0.88
15 Amb-HP	6.3	11	0.54
15 F1-Ax	6.0	12	0.96
15 F1-HP	6.0	13	0.85
15 F2-Ax	5.5	7	0.57
15 F2-HP	6.6	13	0.91
20 Amb-Ax	4.4	22	0.61
20 Amb-HP	2.2	7	0.78
20 F1-Ax	NA	1	NA
20 F1-HP	2.2	4	0.93
20 F2-Ax	2.3	6	0.86
20 F2-HP	3.3	6	0.32

## Discussion

TEP play a key role for aggregation and flux [49] and thus may drive future changes in the functioning of the biological carbon pump [16]. Specifically, it has been hypothesised that increased TEP production under ocean acidification conditions would result in increased aggregation and carbon flux, strengthening the pump [17], [18], although this has also been contested [19]. Under current conditions TEP provide the matrix of marine snow [50], and drive the aggregation of diatoms: Total aggregate volume has been found to be a positive function of total TEP concentration [2], [9], [51], except in the presence of certain minerals (e.g. illite) which promote aggregation [25]. Different scenarios are possible under future conditions of the ocean. Increased TEP production in a future ocean does not necessarily result in increased aggregation, because the stickiness of TEP may also change [19]. Moreover, characteristics of TEP that may change under high *p*CO_2_ and elevated temperatures would impact the packaging of aggregates, and thus their sinking velocities; and consequently flux attenuation and the biological pump. Our experiment, which investigated the response of high temperature and high *p*CO_2_ on TEP formation, aggregation and aggregate sinking velocity allowed us to investigate if the relationships between TEP and aggregation, and that between aggregate size and sinking velocity are likely to change in a future ocean.

### TEP formation

TEP production during the aggregation experiment was a positive function of incubation temperature. Earlier work has also found increased TEP production by diatoms at elevated temperatures [52], [53], but a careful study investigating a larger range of temperatures in eurythermal diatoms found a subsequent decrease when temperatures were increased further [54], suggesting an optimal type response curve. Increased production of TEP at higher temperature is due to increased release of TEP precursors by diatoms [55] and bacteria [56], [57] at higher temperature.

The influence of *p*CO_2_ on TEP production was less clear: At 20°C TEP production peaked under Future 1 conditions, but at 15°C TEP production at Ambient and Future 1 was similar, and no TEP was generated under the Future 2 scenario. As in our experiment, the impact of *p*CO_2_ on TEP production by diatoms has been found to be non-linear in other studies ([25] and references within), with ambiguous results suggesting a complex relationship between TEP-production and *p*CO_2_, possibly dependent on other environmental conditions (light, temperature) or nutrient availability. Significant differences in bacterial catabolism of TEP as a function of environmental conditions would have resulted in differences between treatments with and without *M. adhaerens*, which we did not observe.

The addition of *M. adhaerens* HP15 had no systematic effect on initial TEP concentration, likely because the added volume of acclimatized *M. adhaerens* HP15 cultures was very small. Net TEP production during the aggregation experiment was also not influenced by *M. adhaerens* HP15. Heterotrophic bacteria are known to generate TEP and other EPS, but also utilize it, and their influence on TEP concentrations in the presence of diatoms varies [4]. For example; TEP production by *Thalassiosira rotula* was enhanced by bacteria during exponential phase but reduced during stationary growth of the diatom [3]. *Skeltonema costatum* exhibited a different lifestyle pattern with higher TEP production in the axenic culture compared to the non-axenic one [3]. In an earlier experiment, the presence of *M. adhaerens* HP15 resulted in increased TEP production after 4 and 7 days at 18°C, but TEP was only measured in the surrounding (aggregate-free) seawater [2]. In the experiment presented here TEP concentration in the surrounding seawater was also higher (∼12%) in the presence of *M. adhaerens* HP15, but total TEP concentration was not affected, implying that TEP incorporated in aggregates decreased in the presence of bacteria. Possibly the bacteria impact the fraction of TEP included in aggregates, rather than TEP production *per se*. Conceivably bacterial modification of TEP decreased its propensity to aggregate, or bacterial activity dissociated TEP from aggregates.

### Aggregation

Ranges in size and total volume of aggregates in this experiment were within the ranges found under similar conditions [58], [59], [60]. Aggregation, measured as total aggregated volume, was appreciably higher in all 15°C treatments compared to 20°C treatments. This appears to contradict a study, which found increased aggregation at higher temperatures using a natural diatom population incubated 2.5°C and 8.5°C [53]. The formation of micro-aggregates by the diatom *Skeletonema* sp. in a high turbulence environment was also significantly reduced at 10°C compared to 20°C, but a further increase to 30°C had no effect [61]. However, the carbonate system was not perturbed in either of these studies; and in our experiment the change in temperature had no significant effect on aggregation if only the Ambient treatments are considered. Our study emphasizes that the simultaneous change in temperature and the carbonate system influenced aggregation differently than that of temperature alone: Total aggregate volume was greatly reduced at 20°C under both future *p*CO_2_ scenarios, suggesting synergistic effects between *p*CO_2_ and temperature.

Contrary to expectations, total aggregate volume was not a function of TEP concentration. The lower aggregation in Future 20°C treatments suggests a decreased probability that colliding particles remained attached, called stickiness, in Future 20°C treatments. Aggregation rate is a function of collision rate and stickiness. Collision rate in rolling tanks, where solid body rotation is established, depends largely on particle abundance, size and differential settling [11], [62]. Because cell concentrations, sizes, turbulence and shear, all of which promote collisions, were near identical in all treatments, differences must be a function of TEP or stickiness. As the four treatments with the highest total TEP concentrations resulted in the lowest aggregation, it may be deduced that the average stickiness of particles was lower in Future 20°C treatments compared to the others.

Aggregation was not impacted by the presence or absence of *M. adhaerens* HP15. The impact of heterotrophic bacteria on aggregation can be extremely varied: Bacteria may increase aggregation and stability of aggregates [63], or diatom aggregation may be reduced due to hydrolysis of diatom surface mucus by attached bacteria [3], [64]. Moreover, the role of heterotrophic bacteria for diatom aggregation varies appreciably between algae species and environmental conditions: The presence of bacteria was demonstrated to be a prerequisite for aggregate formation for *T. weissflogii*, but not for *Navicula* sp. [65]. A different study revealed that whether coagulation of *T. rotula* was promoted or reduced in the presence of bacteria depended on light conditions [3]. Earlier work has revealed that the presence of *M. adhaerens* HP15 greatly increased total aggregate volume of *T. weissflogii* [2]. In those experiments, total aggregate volume in the presence of bacteria was 2–3 times higher (10 cm^3^) than in our study, whereas almost no aggregates formed in the axenic cultures. In the present experiment total aggregate volume was independent of the presence of *M. adhaerens* HP15, and total aggregate volume was comparably small. The observed differences in TEP production and aggregation between both experiments were possibly caused by differences in EPS due to experimental (start conditions, time in rolling tank) or cell physiological differences. Composition of extracellular substances released by diatoms varies with growth stage and environmental conditions [66]. Variation in EPS chemistry and tertiary structure between diatom and bacteria EPS are known to result in differences in TEP formation [67], [68], and EPS other than TEP can lead to the formation of aggregates [69]. Moreover, *T.weissflogii* may also aggregate by direct cell to cell attachment [1], and the factors that determine which aggregation process dominates await exploration. The observed discrepancies between similar experiments highlight the complexity (non-linearity) of the bacteria- diatom interactions and aggregation processes under different environmental conditions.

### Sinking velocities of aggregates

The sinking velocity to size relationship of aggregates differed appreciably between treatments, implying differences in aggregate content (excess density) and packaging (porosity) [70]. Sinking velocities of large aggregates formed in 20°C treatments were significantly smaller than those of comparable size formed in 15°C treatments. Sinking velocity is a function of the viscosity of seawater, but this effect should increase sinking velocity at higher temperatures, rather than decrease it [71]. TEP content of aggregates was smaller in the 15°C treatments compared to the 20°C future treatments, as both total TEP concentration and fraction of TEP in aggregates were smaller in 15°C treatments. TEP are positively buoyant and a high proportion of TEP in aggregates reduces their sinking velocity [19], [72], [73]. Differences in chemical composition or quantity of EPS may easily explain differences in packaging and thus sinking velocities. Additionally, differences in the cellular silica content, the biochemical composition of organic matter, or the size of cells [74]–[78] may explain reduced sinking velocity of aggregates consisting of cells grown at higher temperature. Future experiments will need to address these factors.

## Conclusions

The respective roles of TEP, bacteria and diatoms for the formation of diatom aggregates, and the influence of temperature and *p*CO_2_ on coagulation, are complex and not well understood. Differences in environmental conditions and in physiological growth stage of the organisms as well as species specific life strategy differences all contribute to a high variability in the characteristics of the EPS that is produced by diatoms and bacteria or hydrolyzed by bacteria. Our results clearly indicate that the known relationship between TEP and aggregation, and between aggregate size and sinking velocity do not hold under different temperature and *p*CO_2_ scenarios. In contrast to theoretical predictions [17], [18], higher *p*CO_2_ combined with elevated temperatures resulted in increased TEP production, but decreased aggregation and decreased sinking velocity of aggregates, suggesting decreased carbon flux at 1000 m (sequestration flux). Our results thus refute, at least in a general sense, the hypothesis that elevated temperature and ocean acidification as expected in the future ocean will result in increased carbon flux and thus in a negative feed-back to the biological carbon pump [17], [18]. More generally, these results provide an example that established relationships, like that between TEP concentration, aggregation and flux, may not extend to future conditions, and care must be taken, when basing predictions on such empirical relationships.
